# Parasomnias and Associated Factors Among University Students: A Cross-Sectional Study in Saudi Arabia

**DOI:** 10.7759/cureus.48722

**Published:** 2023-11-13

**Authors:** Sultan M Alshahrani, Razan A Albrahim, Jana K Abukhlaled, Lubna H Aloufi, Sarah S Aldharman

**Affiliations:** 1 Neurosciences, King Abdullah Bin Abdulaziz University Hospital, Princess Nourah Bint Abdulrahman University, Riyadh, SAU; 2 College of Medicine, Princess Nourah Bint Abdulrahman University, Riyadh, SAU; 3 College of Medicine, King Saud Bin Abdulaziz University for Health Sciences, Riyadh, SAU

**Keywords:** teeth grinding (bruxism), nocturnal enuresis (sleep enuresis), sleep terrors (night terrors), sleep-related eating disorder (sred), nightmare disorder, rem sleep behavior disorder (rbd), confusional arousals, sleep talking, sleepwalking, parasomnia

## Abstract

Background and aim

Parasomnias are a group of sleep-related movements or emotions like sleepwalking, sleep talking, teeth grinding (Bruxism), nocturnal enuresis (sleep enuresis), sleep terrors (night terrors), sleep-related eating disorder (SRED), nightmare disorder, REM Sleep Behavior Disorder (RBD), and confusional arousals. Parasomnias are more common in children than in adults. This study aimed to estimate the prevalence of different parasomnias among university students in Saudi Arabia. Additionally, it aimed to study the relationship between different parasomnias and gender-associated sleep disorders, mental disorders, and other medical diseases, stress, substance use, and medications.

Methods

This study is a descriptive cross-sectional survey-based study. The target population for this study is university students from different regions of Saudi Arabia. Parasomnia was defined as having at least one of the 11 disorders (over the past six months). Data was collected through an online survey. The survey was distributed on different online platforms to collect data from other regions of Saudi Arabia. The study took place between August and November 2022.

Results

Among 1,296 participants, 934 (72.1%) were female, and 1,071 (82.6%) were aged 19-24 years. A total of 1054 (81, 3%) participants reported having at least one parasomnia disorder. The most prevalent parasomnias were sleep talking 656 (50.6%), nightmares 650 (50.2%), and confusional arousals 524 (40.4%). The least prevalent parasomnia was sleep-related eating disorder 98 (7.6%). Among participants, 580 (44.8%) had a family history of parasomnia, 439 (33.9%) were diagnosed with sleep disorders, 296 (22.8%) were diagnosed with mental illnesses, and 92 (7.1%) had other medical diseases.

Conclusion

Parasomnias are prevalent among university students in Saudi Arabia. Parasomnias were higher in female students and in students with a family history of parasomnia. Parasomnias in adults might be a chronic or recurrent disorder. Parasomnias are significantly associated with psychological stress, depression, and anxiety disorders.

## Introduction

Parasomnias are any irregular or unwanted activity that occurs at the start of sleep, throughout sleep, or during arousals from sleep. These events consist of a wide range of occurrences, including abnormal motor, behavior, and sensory phenomena [1]. The estimated lifetime prevalence of different types of parasomnias rated from 4% to 67% in adults [2,3]. Parasomnias can occur alone or as a comorbid condition with neurological, psychiatric disorders, and trauma. Furthermore, in psychiatric disorders, the parasomnias prevalence rate was noticeably higher. Medication use, other sleep disorders, medical comorbidities, and drug addiction were also reported as risk factors for parasomnias [4].

In relation to the sleep state, these occurrences are frequently categorized into the following two types: rapid eye movement (REM) sleep parasomnias and non-rapid eye movement (NREM) [5]. Parasomnias are classified separately by the Diagnostic and Statistical Manual of Mental Disorders-5 (DSM-5) and the International Classification of Sleep Disorders-3 (ICSD-3).

REM sleep parasomnias compromise REM sleep behavior disorder, recurrent isolated sleep paralysis, and nightmare disorders. Contrarily, NREM parasomnias include behaviors such as confusional arousals, sleepwalking, sleep terrors, and a sleep-related eating disorder. Any stage of sleep might experience additional sorts of parasomnia [6]. NREM parasomnias mostly arise out of slow-wave or deep sleep (N3) of NREM and conditions that cause N3 sleep fragmentation might increase the risk of parasomnias [1]. REM and NREM parasomnias may overlap and occur together in some cases. For the diagnosis of parasomnia, comprehensive sleep history and investigations including polysomnography (sleep study) are important to reach the diagnosis of parasomnia and rule out comorbid conditions. Parasomnias are transit and self-limiting in childhood, in contrast, they can be chronic and debilitating in adults.

Parasomnia disorders unfavorably impact the patient because of substantial disturbed sleep, possible injury, and psychological consequences [1,7]. Gaining more knowledge of parasomnia disorders' characteristics and related features is critical for future diagnosis and management. However, not many studies explore parasomnias in university students; a group of the population identified as being more vulnerable to sleep disorders due to possible increased academic and social expectations, irregular schedules, and other factors [8-10].

In a study conducted to assess the association between sleep dysfunction and psychosis-like experiences among college students, in an ethnically diverse group, 82.5% of participants experienced psychosis-like incidents at least once. College students’ sleep problems included nightmares, night anxiety, non-restorative sleep, fatigue, and initial insomnia [11]. A high prevalence of poor sleep among college students was documented in a multi-university study conducted in the United States. With differences between sexes and mental health symptoms in relation to disturbances. Female participants had a higher prevalence of poor sleep than their male counterparts and were more likely to use sleeping medications. Participants with anxiety symptoms suffered more from sleep disturbance and sleep medication use, whereas participants with depression symptoms were more likely to suffer from increased daytime dysfunction [12]. Our study aimed to find out the prevalence of parasomnia disorder and its associated sleep, mental, and physical factors among university students in Saudi Arabia.

## Materials and methods

Study population

The study is a descriptive cross-sectional survey-based study. Data was collected through an online survey. The survey was distributed on different online platforms to collect data from other regions of Saudi Arabia. The target population for this study is university students from different regions of Saudi Arabia (Central, Southern, Eastern, Western, and North). Parasomnia was defined as having at least one of the 11 disorders (over the past six months); these disorders were listed in the study questionnaire.

Study survey

The study survey was created and data were collected using Google Forms. The survey was distributed randomly by e-mail service at King Abdullah Bin Abdulaziz University Hospital and Princess Nourah Bint Abdulrahman University, as well as through emails and social media groups for university students in different regions in Saudi Arabia.

Statistical analysis and data management

Statistical analysis was carried out using RStudio (R version 4.1.1; Vienna, Austria: R Development Core Team). Categorical variables were expressed as frequencies and percentages. Items with multiple selections were analyzed using a multiple-response analysis. Factors associated with parasomnia were analyzed using univariate binary logistic regression analysis, and the independent risk factors for parasomnia were assessed using a multivariate regression model. Results of the regression analysis were presented as odds ratio (OR) and 95% confidence intervals (95% CIs). A p-value of <0.05 indicated statistical significance.

## Results

Demographic characteristics

A total of 1,300 records were received on the online platform. However, we excluded four records of those who declined to participate. Therefore, the records of 1,296 participants (university students) were analyzed in the current study. Approximately three-quarters of the respondents were females N=934 (72.1%). The majority of respondents were aged 19-24 years N=1,071 (82.6%) and were Saudis N=1,206 (93.1%) and single N=1,155 (89.1%). Residents of the Western and Central Region represented N=303 (23.4%) and N=299 (23.1%) of the sample, respectively, and the majority of students were living with their families N=1,120 (86.4%). Students of the first and sixth academic years were the most frequently participating students N=262 (20.2%) and N=251 (19.4%), respectively. Only N=166 (12.8%) of students were smokers (Table 1).

**Table 1:** Demographic characteristics of the participants. Data are presented as N (%).

Parameter	Category	N (%)
Age (year)	19-24	1,071 (82.6%)
	25-30	165 (12.7%)
	31-35	28 (2.2%)
	>35	32 (2.5%)
Gender	Male	362 (27.9%)
	Female	934 (72.1%)
Nationality	Saudi	1,206 (93.1%)
	Non-Saudi	90 (6.9%)
Region of residence	Northern region	296 (22.8%)
	Southern region	281 (21.7%)
	Eastern region	117 (9.0%)
	Western region	303 (23.4%)
	Central region	299 (23.1%)
Marital status	Single	1,155 (89.1%)
	Married	130 (10.0%)
	Divorced	11 (0.8%)
Academic level	1st year	262 (20.2%)
	2nd year	237 (18.3%)
	3rd year	207 (16.0%)
	4th year	227 (17.5%)
	5th year	112 (8.6%)
	6th year	251 (19.4%)
Place of residence	With family	1,120 (86.4%)
	Student housing	124 (9.6%)
	Other	52 (4.0%)
Smoker	Yes	166 (12.8%)

Prevalence and characteristics of parasomnia

In general, N=1,054 participants reported having at least one parasomnia disorder, representing (81.3%) of the overall sample (95% CI: 79.1-83.4). The most frequent parasomnia disorders that had been reported over the past six months included talking during sleep N=656 (50.6%), nightmares N=650 (50.2%), and waking up at night in a confused state without remembering the event the next day N=524 (40.4%). Whereas the least reported parasomnia was eating food in sleep or at night in a room or somewhere else without realizing it N=98 (7.6%). A total of N=580 (44.8%) students have a family history of parasomnia disorders (Table 2).

**Table 2:** Participants’ responses to parasomnia disorders. Data are presented as N (%)

Parameter	No	Yes
Experienced or been told that you have been sleepwalking	1,129 (87.1%)	167 (12.9%)
Experienced or been told that you have talked in your sleep	640 (49.4%)	656 (50.6%)
Experienced or been told that you have woken up at night in a confused state without remembering the event the next day	772 (59.6%)	524 (40.4%)
Experienced or been told that you have injured yourself during sleep	1,156 (89.2%)	140 (10.8%)
Experienced or been told that you have injured somebody else during sleep	1,172 (90.4%)	124 (9.6%)
Experienced nightmares in your sleep	646 (49.8%)	650 (50.2%)
Experienced or been told that you have eaten food in your sleep or at night in your room or somewhere else without realizing it	1,198 (92.4%)	98 (7.6%)
Experienced or been told that you have woken up at night in terror screaming without remembering the event the next day	1,000 (77.2%)	296 (22.8%)
Experienced or been told that you grind your teeth while sleeping	1,145 (88.3%)	151 (11.7%)
Ever involuntarily urinated while asleep	976 (75.3%)	320 (24.7%)
Ever experienced or been told that something else not mentioned above has happened to you while you were sleeping in the past six months	1,186 (91.5%)	110 (8.5%)
Has anyone in your family or your relatives experienced anything mentioned above during their sleep?	716 (55.2%)	580 (44.8%)

Characteristics of sleep, mental, and physical disorders

Generally, almost one-third of students had been diagnosed with a sleep disorder N=439 (33.9%). Whereas N=296 (22.8%) and N=92 (7.1%) of them had been diagnosed with a mental illness or a medical disease, respectively. Additionally, N=184 (14.2%) of students were receiving a medication. Substance use was only present in N=25 (1.9%) of the participants. Self-reported psychological stress was apparent among N=906 (69.9%) of students, of whom N=181 (20.0%), N=442 (48.8%), and N=220 (24.3%) reported minimal, moderate, or severe effects of stress on sleep, respectively (Table 3).

**Table 3:** Characteristics of sleep, mental, and physical disorders. *Descriptive data is based on 906 students who had psychological stress. Data are presented as N (%)

Parameter	Category	N (%)
Diagnosed with a sleep disorder	No	857 (66.1%)
	Yes	439 (33.9%)
Diagnosed with a mental disorder	No	1,000 (77.2%)
	Yes	296 (22.8%)
Diagnosed with a medical disease	No	1,204 (92.9%)
	Yes	92 (7.1%)
Take any medication	No	1,112 (85.8%)
	Yes	184 (14.2%)
Substance use	No	1,271 (98.1%)
	Yes	25 (1.9%)
Suffer from any psychological stress from college, home, or others	No	390 (30.1%)
	Yes	906 (69.9%)
The effect of stress on sleep*	No effect	63 (7.0%)
	Minimally	181 (20.0%)
	Moderately	442 (48.8%)
	Severely	220 (24.3%)

Factors associated with parasomnia

Based on the univariate analysis, parasomnia was significantly lower among students aged 25-30 years (OR=0.54, 95% CI: 0.37-0.79, p=0.001) and more than 35 years (OR=0.44, 95% CI: 0.21-1.00, p=0.038), as well as the students in the sixth academic year (OR=0.53, 95% CI: 0.34-0.82, p=0.004). Conversely, parasomnia was significantly higher among females (OR=1.63, 95% CI: 1.21-2.18, p=0.001), as well as being diagnosed with a sleep disorder (OR=2.61, 95% CI: 1.86-3.74, p<0.001), a mental disorder (OR=4.18, 95% CI: 2.64-7.03, p<0.001), and having psychological stress (OR=3.05, 95% CI: 2.29-4.07, p<0.001) (Table 4).

**Table 4:** Factors associated with having symptoms of parasomnia. Data are presented as odds ratios (ORs) and 95% confidence intervals (CIs). Statistical significance is set at p<0.05.

Parameter	Category	Univariate			Multivariate		
		OR	95% CI	p-Value	OR	95% CI	p-Value
Age	19-24	-	-	-	-	-	-
	25-30	0.54	0.37, 0.79	0.001	0.76	0.48, 1.21	0.240
	31-35	0.61	0.27, 1.56	0.259	1.02	0.41, 2.80	0.975
	>35	0.44	0.21, 1.00	0.038	0.57	0.25, 1.37	0.191
Gender	Male	-	-	-	-	-	-
	Female	1.63	1.21, 2.18	0.001	1.23	0.89, 1.70	0.209
Nationality	Saudi	-	-	-	NA	NA	NA
	Non-Saudi	0.74	0.45, 1.25	0.241	NA	NA	NA
Region of residence	Northern region	-	-	-	NA	NA	NA
	Southern region	0.84	0.56, 1.27	0.405	NA	NA	NA
	Eastern region	0.80	0.48, 1.37	0.402	NA	NA	NA
	Western region	1.13	0.74, 1.72	0.575	NA	NA	NA
	Central region	1.14	0.75, 1.74	0.552	NA	NA	NA
Marital status	Single	-	-	-	NA	NA	NA
	Married	0.77	0.50, 1.21	0.242	NA	NA	NA
	Divorced	0.39	0.12, 1.49	0.132	NA	NA	NA
Academic level	1st year	-	-	-	-	-	-
	2nd year	0.84	0.52, 1.34	0.457	0.76	0.47, 1.25	0.283
	3rd year	0.91	0.56, 1.50	0.713	0.94	0.56, 1.58	0.806
	4th year	1.02	0.62, 1.67	0.944	0.95	0.57, 1.60	0.855
	5th year	0.72	0.41, 1.28	0.252	0.67	0.37, 1.24	0.195
	6th year	0.53	0.34, 0.82	0.004	0.67	0.40, 1.13	0.133
Place of residence	With family	-	-	-	NA	NA	NA
	Student housing	1.06	0.67, 1.77	0.803	NA	NA	NA
	Other	0.86	0.45, 1.77	0.653	NA	NA	NA
Smoker	No	-	-	-	NA	NA	NA
	Yes	1.05	0.70, 1.62	0.832	NA	NA	NA
Sleep disorder	No	-	-	-	-	-	-
	Yes	2.61	1.86, 3.74	<0.001	1.86	1.29, 2.72	0.001
Mental disorder	No	-	-	-	-	-	-
	Yes	4.18	2.64, 7.03	<0.001	2.55	1.56, 4.39	<0.001
Medical disease	No	-	-	-	-	-	-
	Yes	2.21	1.16, 4.79	0.027	1.71	0.85, 3.83	0.158
Substance use	No	-	-	-	NA	NA	NA
	Yes	0.92	0.37, 2.78	0.864	NA	NA	NA
Take any medications	No	-	-	-	NA	NA	NA
	Yes	1.33	0.88, 2.08	0.195	NA	NA	NA
Psychological stress	No	-	-	-	-	-	-
	Yes	3.05	2.29, 4.07	<0.001	2.53	1.87, 3.43	<0.001

Risk factors for parasomnia

The significantly associated factors from the univariate regression analysis were used as independent variables in a multivariate binary logistic regression model to account for the independent associations with parasomnia. Results indicated that three independent risk factors, including having psychological stress (OR=2.53, 95% CI: 1.87-3.43, p<0.001), being diagnosed with a sleep disorder (OR=1.86, 95% CI: 1.29-2.72, p=0.001), and a mental disorder (OR=2.55, 95% CI: 1.56-4.39, p<0.001) (Table 4).

The association between the symptoms of parasomnia and having a confirmed diagnosis of sleep, mental, and physical conditions

In order to further assess the association between the symptoms of parasomnia and having a confirmed diagnosis of sleep, mental, and physical conditions, results showed that parasomnia symptoms were significantly associated with having a confirmed diagnosis of parasomnia (OR=7.79, 95% CI: 3.24-25.6, p<0.001), insomnia (OR=2.15, 95% CI: 1.52-3.11, p<0.001), obstructive sleep apnea (OR=5.48, 95% CI: 1.68-33.7, p=0.019), depression (OR=4.46, 95% CI: 2.38-9.55, p<0.001), and anxiety (OR=3.63, 95% CI: 2.11-6.81, p<0.001) (Table 5). However, on the multivariate analysis adjusted for demographic variables (age, gender, nationality, region of residence, marital status, academic level, place of residence, and smoking status), parasomnia symptoms were significantly predicted by having a confirmed diagnosis of parasomnia (OR=4.86, 95% CI: 1.97-16.2, p=0.003), insomnia (OR=1.48, 95% CI: 1.02-2.20, p=0.045), and depression (OR=2.97, 95% CI: 1.52-6.51, p=0.003) (Table 6).

**Table 5:** The association between the symptoms of parasomnia and having a confirmed diagnosis of sleep, mental, and physical conditions. Data are presented as odds ratios (ORs) and 95% confidence intervals (CIs). Statistical significance is set at p<0.05.

Domain	Parameter	Category	OR	95% CI	p-Value
Sleep disorders	Parasomnia	No	-	-	-
		Yes	7.79	3.24, 25.6	<0.001
	Insomnia	No	-	-	-
		Yes	2.15	1.52, 3.11	<0.001
	Obstructive sleep apnea	No	-	-	-
		Yes	5.48	1.68, 33.7	0.019
	Other sleep disorders	No	-	-	-
		Yes	1.38	0.23, 26.1	0.766
Mental disorders	Depression disorder	No	-	-	-
		Yes	4.46	2.38, 9.55	<0.001
	Anxiety disorder	No	-	-	-
		Yes	3.63	2.11, 6.81	<0.001
	Obsessive-compulsive disorder	No	-	-	-
		Yes	0.34	0.06, 2.61	0.242
	PTSD	No	-	-	-
		Yes	2.25	0.79, 9.47	0.183
	Bipolar disorder	No	-	-	-
		Yes	NA	NA	0.975
	Other mental disorders	No	-	-	-
		Yes	NA	NA	0.979
Medical disorders	Brain tumor	No	-	-	-
		Yes	NA	NA	0.974
	Seizure disorder	No	-	-	-
		Yes	NA	NA	0.975
	Genetic disorders	No	-	-	-
		Yes	2.78	0.54, 50.7	0.327
	Traumatic brain injury	No	-	-	-
		Yes	NA	NA	0.978
	Multiple sclerosis	No	-	-	-
		Yes	0.46	0.04, 9.88	0.525
	Scoliosis	No	-	-	-
		Yes	NA	NA	0.973
	Others	No	-	-	-
		Yes	1.15	0.54, 2.86	0.735
Substance use	Alcohol	No	-	-	-
		Yes	0.92	0.29, 4.05	0.894
	Cannabis	No	-	-	-
		Yes	1.15	0.18, 22.1	0.899
	Others	No	-	-	-
		Yes	0.69	0.09, 13.9	0.746
	Amphetamine	No	-	-	-
		Yes	NA	NA	0.975
	Methamphetamine	No	-	-	-
		Yes	0.46	0.04, 9.88	0.525

**Table 6:** Results of the multivariate, adjusted regression analysis for the predictors of having parasomnia symptoms among students. Data are presented as odds ratios (ORs) and 95% confidence intervals (CIs). Statistical significance is set at p<0.05.

Parameter	Category	OR	95% CI	p-Value
Parasomnia	No	-	-	-
	Yes	4.86	1.97, 16.2	0.003
Insomnia	No	-	-	-
	Yes	1.48	1.02, 2.20	0.045
Obstructive sleep apnea	No	-	-	-
	Yes	3.19	0.92, 20.2	0.120
Depression disorder	No	-	-	-
	Yes	2.97	1.52, 6.51	0.003
Anxiety disorder	No	-	-	-
	Yes	1.79	0.98, 3.50	0.071

## Discussion

Our study assessed a total of N=1,296 participants, N=934 females (72.1%) and N=362 males (27.9%). Most of the participants N=1,071 (82.6%) are young adults (aged 19-24 years). Among participants, N=1054 (81, 3%) reported having at least one parasomnia disorder. The most prevalent parasomnias over the past six months are sleep talking N=656 (50.6%), nightmare disorder N=650 (50.2%), and unexpectedly waking up at night in a confused state without remembering the event the next day (confusional arousals) in N=524 (40.4%) students. Whereas the least reported disorder was eating food in sleep or at night in a room or somewhere else without realizing it (sleep-related eating disorder) in N=98 students (7.6%). Our study also found surprisingly N=320 students (24.7%) have nocturnal enuresis and N=296 students (22.8%) have sleep terrors. More importantly, N=140 (10.8%) and N=124 participants (9.6%) have experienced or been told that they have injured themselves or somebody else, respectively, during sleep, which might represent REM Sleep Behavior Disorder (RBD), but we didn’t ask further questions in this regard.

To the best of our knowledge, the prevalence of parasomnias among adults in Saudi Arabia was not well studied, and this is the first study specifically aiming to find the prevalence of parasomnias among adults in Saudi Arabia. Most studies in Saudi Arabia looked at obstructive sleep apnea, insomnia, and sleep quality among adult population. Regarding parasomnia, some studies looked at some parasomnias as part of sleep habits in children in Saudi Arabia. Few studies in Saudi Arabia mentioned parasomnia prevalence as part of sleep disorders in general. A cross-sectional study in Makkah City in Saudi Arabia found that the prevalence of sleepwalking was 3.7% and nightmares was 13.7% among medical students [13].

Globally, many researchers studied the prevalence of parasomnia among adults. The estimated lifetime prevalence of the different parasomnias varied from about 4% to 67% [2,3]. For sleep walking lifetime prevalence was 22.4% and current prevalence was 1.7%. For the other parasomnias, lifetime and current prevalence were as follows: sleep talking 66.8% and 17.7%, confusional arousals 18.5% and 6.9%, sleep terrors 10.4% and 2.7%, injured yourself during sleep 4.3% and 0.9%, injured somebody else during sleep 3.8% and 0.4%, sexual acts during sleep 7.1% and 2.7%, nightmare 66.2% and 19.4%, dream enactment 15.0% and 5.0%, sleep-related groaning 31.3% and 13.5%, and sleep-related eating 4.5% and 2.2% [2,3,14].

In our study, we found an unexpectedly high prevalence of nocturnal enuresis in N=320 (24.7%) students and sleep terrors N=296 (22.8%). However, in adults globally, the reported prevalence of enuresis is 0.5-2% [15-25]. Sleep terrors in adults are extremely rare [1]. A Korean epidemiologic study found that the overall prevalence of nocturnal enuresis in subjects aged 16-40 years was 2.6% [26]. The expected explanation could be that some participants thought of the question as nocturia, which is defined as waking up from sleep to urinate. The other explanation is that some students have urological symptoms that may cause frequent urination during the night.

In our study, we found the estimated prevalence of REM Sleep Behavior Disorder (RBD) was N=140 (10.8%) and N=124 (9.6%) who have injured themselves or somebody else, respectively, during sleep. However, the prevalence of RBD globally is estimated at 0.5-1.25% in the general population, with higher frequencies (2%) among older adults and those with Parkinson's disease (PD), multiple system atrophy, and dementia with Lewy bodies [27-30]. Though REM Sleep Behavior Disorder (RBD) prevalence is still globally underdiagnosed and might be missed in many cases, it was higher than expected in our study. This could be explained by misunderstanding of our questions by participants ("Experienced or been told that you have injured yourself during sleep?" and "Experienced or been told that you have injured somebody else during sleep?"). The other explanation may be that participants recorded any injurious behavior due to other NREM parasomnias like sleepwalking as REM-related. Moreover, participants in our study have other risk factors for REM Sleep Behavior Disorder (RBD) like antidepressant medications or alcohol and other substance use.

The second objective of our research is to study the relationship between different parasomnias and gender-associated sleep disorders, family history, mental disorders, stress, medical diseases, medications, smoking, and substance abuse. We have found that parasomnias were significantly reported more prevalent in female students. A study found that parasomnias are more prevalent at a young age and in female gender [3]. Nielsen and Zadra in 2005 found that nightmares were more common in the female population [31]. However, studies did not find significant gender differences in parasomnias [2]. Moreover, REM Sleep Behavior Disorders (RBD) are reported to be more common in male patients who are over the age of 50 years, but our study population was younger.

In our study, we found that N=580 (44.8%) students had a family history of parasomnia disorders. Studies found that first-degree relatives of patients with parasomnias have a higher prevalence of similar parasomnias [1]. There is often a high prevalence of nocturnal enuresis (sleep enuresis) among the parents, siblings, and other relatives of children with primary enuresis [1]. Kales et al. in 1980 found that patients with sleep terrors and sleepwalking have a family history of similar disorders [32]. Lopez et al. in 2013 found that 56.6% of sleepwalkers and 57.9% with violent sleep-related behaviors have a family history of sleepwalking [33]. It became known that parasomnias are widely recognized to have a family history without a clear mode of genetic transmission. However, few studies pointed to the genetic background for parasomnias and concluded that NREM parasomnias share a common genetic predisposition [34]. Licis et al. in 2011 found that sleepwalking may be transmitted as an autosomal dominant trait at chromosome 20 [35]. However, no further studies have supported these findings or denied them.

Our study also found that parasomnias are highly associated with previous diagnoses of sleep disorders in participants namely parasomnia and insomnia, N=439 (33.9%). This finding indicates that parasomnias in adults might be chronic or recurrent disorders due to certain factors like psychological stress and psychiatric disorders like depression and anxiety. The relation between parasomnias and insomnia can be explained as comorbidity, however, this comorbidity was not well studied [36]. Conversely, this relation can be reciprocal or bidirectional meaning that it can go both ways. Insomnia, use of hypnotics, and conditions that cause increased sleep drive like sleep deprivation are risk factors for parasomnias [1]. Moreover, some parasomnias can cause sleep interruption and non-refreshing sleep [3]. Furthermore, a study found significant associations between sleepwalking and insomnia, daytime sleepiness, and fatigue [33].

Our study has also found that parasomnias were highly associated with existing psychological stress defined by our participants, N=906 (69.9%). The association of stress with parasomnias has not been well studied in previous studies. However, post-traumatic stress disorder (PTSD) can cause REM Sleep Behavior Disorder (RBD) and nightmare disorder [37,38]. Acute stress is considered a precipitating factor in sleep-related eating disorder (SRED) [1].

Finally, our study found that parasomnias are highly associated with previous diagnoses of mental disorders like depression and anxiety disorders, N=296 (22.8%). Previous studies found that depressive mood was associated with confusional arousals, sleep terrors, sleep-related injury, and nightmare disorder [3]. Another study reported a significantly higher prevalence rate of parasomnia in psychiatric conditions, with nightmares being 38.9%, sleep paralysis 22.3%, sleep-related eating disorders 9.9%, sleepwalking 8.5%, and RBD 3.8% [4]. Moreover, Lopez et al. in 2013 found significant associations between sleepwalking, depression, and anxiety [33]. Furthermore, a study showed that nightmare disorders were significantly associated with mood disorders [39]. As we have mentioned earlier that sleep terrors in adults are rare, but if happen, they are more likely to be associated with medications or psychopathology [1].

Then, we studied the association of parasomnias with medical diseases medications, smoking, and substance abuse. We did not find a strong association between parasomnias and these variables in our sample. Our study participants are healthy young university students, this might explain the lack of organic diseases and medication use, but it does not definitely explain smoking and substance use because they might increase relatively in such population. However, several studies have found this association in different populations. Studies found an association between sleepwalking and other disorders like sleep apnea, RLS, and hypnotic medications [1]. Medications like benzodiazepine receptor agonists, antidepressants like (amitriptyline, paroxetine, mirtazapine, and bupropion), antipsychotics like (olanzapine and quetiapine), antihypertensives like (propranolol and metoprolol), fluoroquinolones, montelukast, and topiramate are triggers for sleepwalking [28,40]. Additionally, confusional arousals can occur in adults with sedative-hypnotic use [1]. Sleep-related eating disorder (SRED) might be caused by medications like zolpidem, smoking cessation, and alcohol consumption [1]. The research found three factors contribute to the risk of nocturnal enuresis (sleep enuresis) in adults; they are large nocturnal urine volume production, nocturnal bladder over-activity, and difficulty arousing from sleep [1]. A study found that medication use, other sleep disorders, medical comorbidities, and drug addiction were also reported as risk factors for parasomnias [4].

Studies found that REM Sleep Behavior Disorder (RBD) has been associated with different neurological disorders, antidepressant medications, and alcohol [1]. Recent studies found a strong association between RBD Parkinson’s disease and neurodegenerative α-synucleinopathy [1,41-43]. RBD can occur in pontine tegmental lesions, alcohol withdrawal, antidepressant medications like selective serotonin reuptake inhibitors (SSRIs), serotonin-norepinephrine reuptake inhibitors (SNRIs), tricyclic antidepressants (TCAs), and monoamine oxidase inhibitors (MAOIs), narcolepsy, and other neurodegenerative disorders like frontotemporal dementia and Alzheimer’s dementia [1,41,44]. A parasomnia identified as sleep-related hallucinations (visual hallucinations that occur at the onset of sleep or upon awakening from sleep) is seen in neurological disorders like Charles Bonnet syndrome and peduncular hallucinosis [1,45,46].

Limitations

Our study is a cross-sectional study which has some limitations. These limitations include (i) inability to measure the incidence, (ii) difficulty to make a causal inference, and (iii) associations identified that might be difficult to interpret. Additionally, the online distribution of the questionnaire might affect the representativeness of the study sample. Nonetheless, based on recent data from the World Bank Database, approximately 98% of the Saudi population uses the internet, which implies it is widely available and readily accessible [47].

## Conclusions

Parasomnias are prevalent among university students in Saudi Arabia. Parasomnias were high in female students and in students with a family history of parasomnia. Parasomnias in adults might be a chronic or recurrent disorder. Parasomnias are significantly associated with psychological stress, depression, and anxiety disorders. Prospective studies should be conducted to investigate the prevalence of parasomnias and their associated factors in Saudi Arabia and worldwide.
